# Development of a core outcome set based on Case Report Form (CRF) to assess laboratory biomarkers and clinical parameters in Onco-Hematology area

**DOI:** 10.1186/1745-6215-16-S1-P23

**Published:** 2015-05-29

**Authors:** Mariangela Vanalli, Francesca Rio

**Affiliations:** 1 Department of Immunohematology and Transfusion Medicine, Hospital Papa Giovanni XXIII, Bergamo, Italy; 2 University of Milan Bicocca, Milan, Italy

## Background

The number of cases, the crude and age-standardized incidence, mortality rates and the prevalence proportions estimated by the Italian Association of Cancer Registries (AIRTUM) presently providing the epidemiological indicators for the major cancers used in ICD-O-3.1 [1-3]. By 2012, the breast cancer incidence in women (age 25±over 85 years) was about 29%; trends for stomach and colorectal cancer were about 5% and 14% for both genders (age 35/45±over 85 years); the lung cancer incidence rates was about 15% in men (age 45±over 85 years) and 6% in women (age 40±over 85) in 2009 [4,5]. From 2011 onwards the tendency changed: the female rates (20 per 100,000) increased much more rapidly than the male rates [6].

Aim of this study is to examine the relationships among the incidence of genera-cancer-associated risk factors and routine laboratory in cancer patients through CRF.

## Materials and Methods

**Fig 1:**
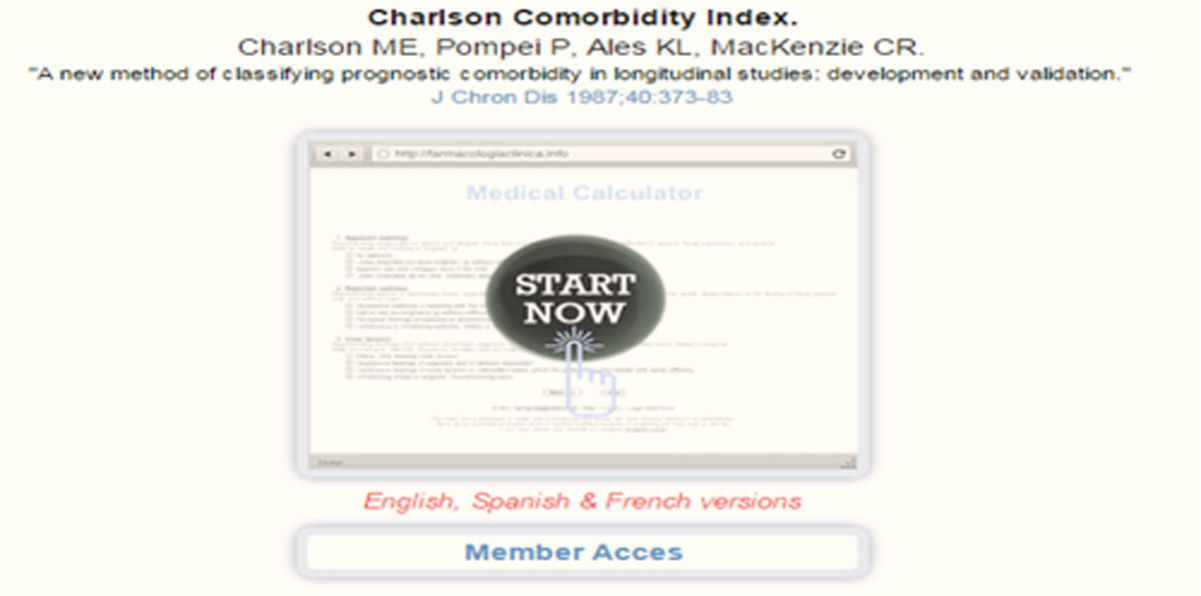
Charlson Comorbidity Index Acces.

The CRF database has been developed by a dedicated working group using Delphi process. It contain anonymous records on patient characteristics (gender, age, alcohol and smoking history, height, body weight, performance status measured using the Eastern Cooperative Oncology Group-ECOG PS, chronic comorbidities weighted by the Charlson Comorbidity Index-CCI, type and stage of tumor) (Figure 1) [7-9] and one set of biomarker laboratory data identified in several variables (Table 1) [10,11].

**Table 1:** Multivariate Analyses of cancer type, comorbidity score and biomarkers laboratory

Comorbidity	Breast p	Colon-rectum p	Stomach p	Lung p
HCT_cod	0.105	0.708	0.387	0.078
Hb_cod	0.035	0.775	0.466	0.351
RBC_cod	0.564	0.343	0.194	0.448
WBC_cod	0.292	0.172	0.930	0.583
PLT_cod	0.167	0.535	0.401	0.332

**CCI_SCORE ≥4**	**0.495**	**0.029**	**0.092**	**0.381**

cancer type: breast, colon-rectum, stomach and lung; biomarkers laboratory: HCT, Hb, RBC, WBC, PLT;

comorbidity: CCI_SCORE **≥4**.

## Results

**Fig 2:**
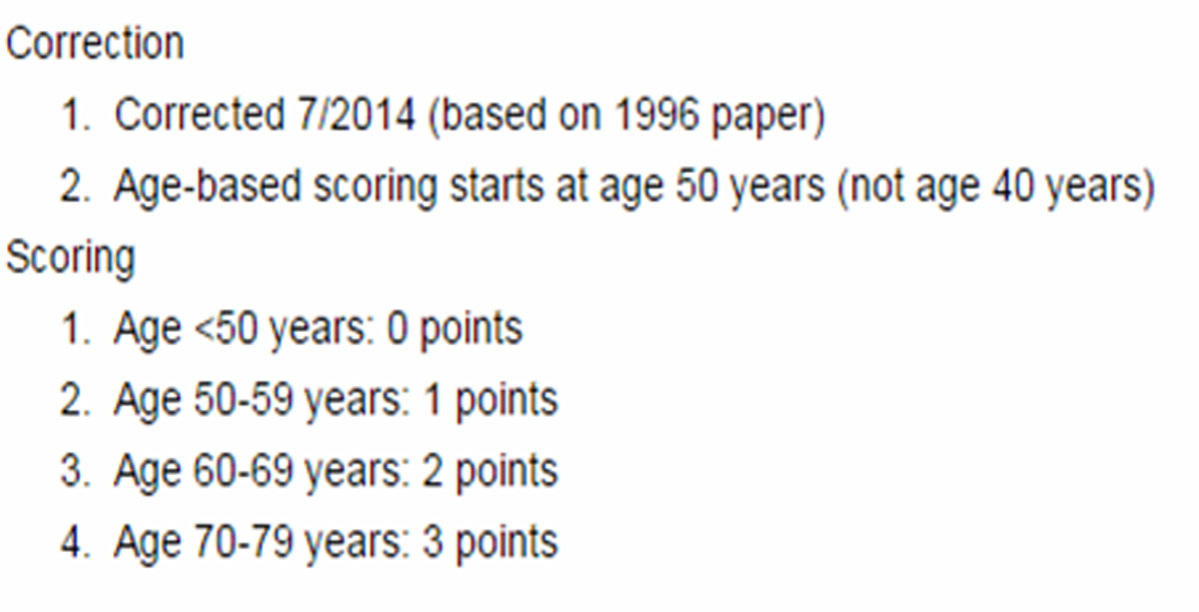
CCI and their respective point scores.

**Fig 3:**
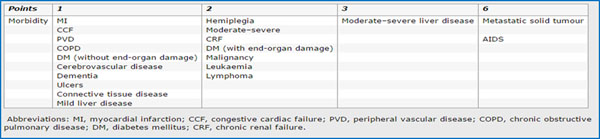
CCI and their respective point scores.

**Fig 4:**
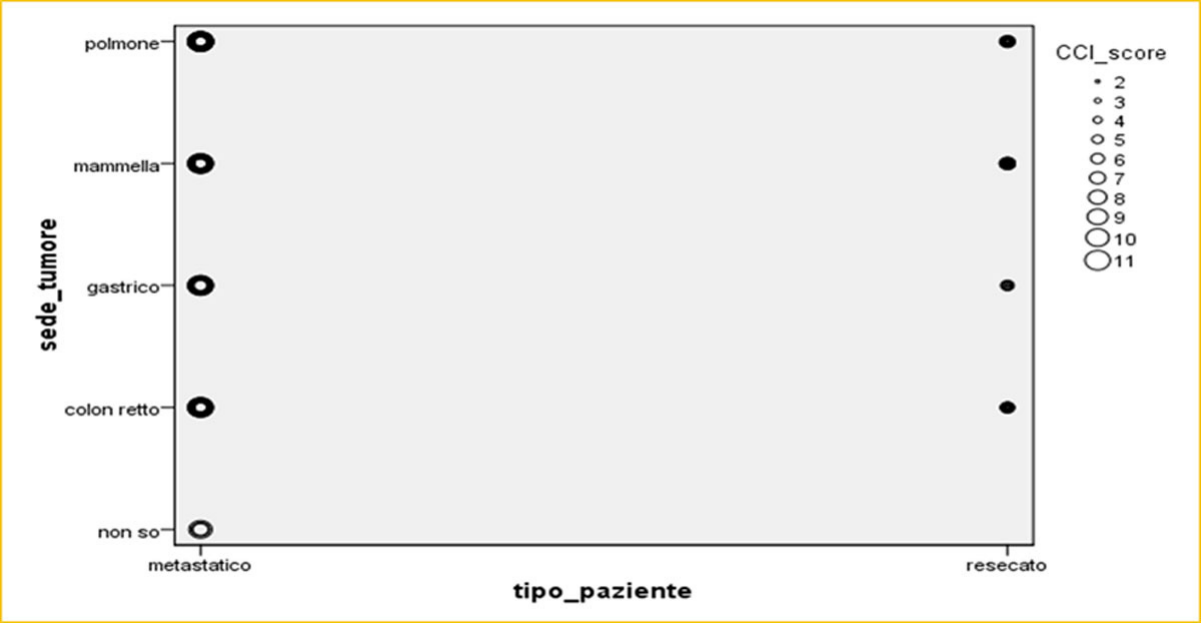
Multivariate Analysis and the comorbidities of CCI with IBM SPSS Italian version 21 statistical software.

**Fig 5:**
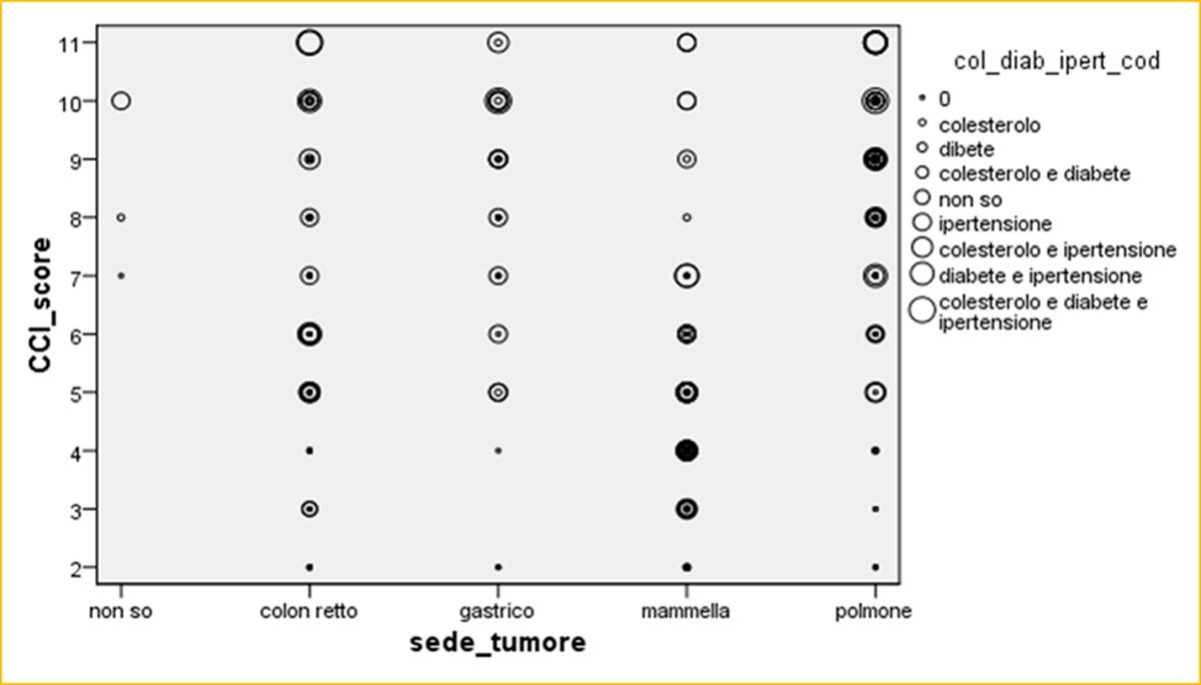
Multivariate Analysis and the comorbidities of CCI with IBM SPSS Italian version 21 statistical software.

Between 2012 and 2014, 1373 cancer patients were enrolled at three Italian Oncological Institutions after informed consent. Among these patients, 36% were men and 64% were women (mean age 71±45 years) (Figure 2) and breast was the most frequent type cancer (43%) followed by lung (29%), colon-rectum (18%) and stomach (9%). 72% (n=85) of the lung, 67% (n=24) of the stomach, 33% (n=25) of the colon-rectum, 4% (n=7) of the breast cancer patients had comorbidities weighted with 3 point and above (Age Unadjusted Charlson-Comorbidity-Index≥4; HR=6.38; 99% CI [3.07,13.24]) [12,13] (Figure 3). Multivariate analysis determined that comorbidity was highly associated with cancer type, stage and ECOG PS (p=0.01) (Figure 4). Evaluation between cardiovascular disease, risk of bleeding, deep-vein thrombosis and colon-rectum cancer stage (p=0.01), breast (p=0.03), lung (p=0.01) compared into comorbidities (Figure 5). The other tested variables: Hgb level, neutrophil and platelet counthad had the strongest relationship with breast, lung cancer stage (p=0.02), stomach (p=0.002) and colon-rectum (p=0.1) [14,15].

## Conclusions

The appropriateness of results could be useful to better describe the role of CRF and biomarkers recorded in patient charts as well as the other variables could allow nurses to identify patients at risk for shorter survival time following hospitalization [16,17].

## References

[B1] Abbasi S Badheeb A Prognostic factors in advanced non-small-cell lung cancer patients: Patient characteristics and type of chemotherapy Lung Cancer International 2011 0 0 1 4 10.4061/2011/152125 PMC4475580 26316930

[B2] Albain KS Crowley JJ LeBlanc M Livingston RB Survival determinants in extensive-stage non-small-cell lung cancer: The Southwest Oncology Group experience Journal of Clinical Oncology 1991 9 1618 1626 1651993 10.1200/JCO.1991.9.9.1618

[B3] American Cancer Society Cancer facts and figures 2012 Retrieved from [http://www.cancer.org/acs/groups/content/@epidemiologysurveilance/documents/document/acspc-031941.pdf]. Accessed November 21, 2014

[B4] Boyd CM Vollenweider D Puhan MA Informing evidence-based decision-making for patients with comorbidity: availability of necessary information in clinical trials for chronic diseases PLoS ONE 2012 7 e41601 10.1371/journal.pone.0041601 22870234 PMC3411714

[B5] Charlson ME Pompei P Ales KL MacKenzie CR A new method of classifying prognostic comorbidity in longitudinal studies: Development and validation J Chronic Dis 1987 40 5 373 383 10.1016/0021-9681(87)90171-8 3558716

[B6] Corbett J Laboratory tests and diagnostic procedures with nursing diagnoses 2008 7 Upper Saddle River, NJ: Pearson/Prentice Hall

[B7] Cox D Oakes D Analysis of survival data 1984 London, England: Chapman and Hall

[B8] Foucher ES O’Callaghan M Ferlay J Masuyer E Rosso S Forman D Bray F Comber H The European Cancer Observatory: A new data resource European Journal of Cancer 2014 0 0 1 13 10.1016/j.ejca.2014.01.027 24569102

[B9] Gatta G Ciampichini R Bisanti L Contiero P Tessandori R Baili P Rossi S Estimates of cancer burden in Lombardy Tumori 2013 99 3 277 84 24158056 10.1177/030089161309900302

[B10] Grande E Inghelmann R Francisci S Verdecchia A Micheli A Baili P Capocaccia R De Angelis R Regional estimates of all cancer malignancies in Italy Tumori 2007 93 345 351 17899864 10.1177/030089160709300404

[B11] Jemal A Siegel R Ward E Hao Y Xu J Thun MJ Cancer statistics A Cancer Journal for Clinicians 2009 59 4 225 249 10.3322/caac.20006 19474385

[B12] International Agency for Research on Cancer Cancer Incidence in Five Continents Annual Dataset 2014 http://ci5.iarc.fr/CI5plus/ci5plus.htm Accessed November 21

[B13] Luo J Chen YJ Narsavage GL Ducatman A Predictors of Survival in Patients with Non-Small Cell Lung Cancer Oncology Nursing Forum 2012 39 6 609 16 10.1188/12.ONF.609-616 23107855

[B14] Oken MM Creech RH Tormey DC Horton J Davis TE Mc-fadden ET Carbone PP Toxicity and response criteria of the Eastern Cooperative Oncology Group American Journal of Clinical Oncology 1982 5 6 649 655 10.1097/00000421-198212000-00014 7165009

[B15] Quan H Li B Couris CM Fushimi K Graham P Hider P Januel JM Sundararajan V Updating and validating the Charlson comorbidity index and score for risk adjustment in hospital discharge abstracts using data from 6 countries Am J Epidemiol 2011 173 676 82 10.1093/aje/kwq433 21330339

[B16] Radovanovic D Seifert B Urban P Eberli FR Rickli H Bertel O Puhan MA Erne P Validity of Charlson Comorbidity Index in patients hospitalised with acute coronary syndrome. Insights from the nationwide AMIS Plus registry 2002-2012 Heart 2014 100 4 288 94 [ClinicalTrials.gov Identifier NCT01305785] 10.1136/heartjnl-2013-304588 24186563

[B17] Yurkovich M Zubieta JAA Thomas J Gorenchtein M Lacaille D A systematic review identifies valid comorbidity indices derived from administrative health data Journal of Clinical Epidemiology 2015 68 3 14 10.1016/j.jclinepi.2014.09.010 25441702

